# The Relationship Between Smartphone-Recorded Environmental Audio and Symptomatology of Anxiety and Depression: Exploratory Study

**DOI:** 10.2196/18751

**Published:** 2020-08-13

**Authors:** Daniel Di Matteo, Kathryn Fotinos, Sachinthya Lokuge, Julia Yu, Tia Sternat, Martin A Katzman, Jonathan Rose

**Affiliations:** 1 The Centre for Automation of Medicine The Edward S Rogers Sr Department of Electrical and Computer Engineering University of Toronto Toronto, ON Canada; 2 Stress Trauma Anxiety Rehabilitation Treatment Clinic for Mood and Anxiety Disorders Toronto, ON Canada; 3 Department of Psychology Adler Graduate Professional School Toronto, ON Canada; 4 Department of Psychology Lakehead University Thunder Bay, ON Canada; 5 The Northern Ontario School of Medicine Thunder Bay, ON Canada

**Keywords:** depression, anxiety, mobile phone, ecological momentary assessment, mobile apps, mobile health, digital signal processing, acoustics, speech recognition software

## Abstract

**Background:**

*Objective* and *continuous* severity measures of anxiety and depression are highly valuable and would have many applications in psychiatry and psychology. A collective source of data for objective measures are the sensors in a person’s smartphone, and a particularly rich source is the microphone that can be used to sample the audio environment. This may give broad insight into activity, sleep, and social interaction, which may be associated with quality of life and severity of anxiety and depression.

**Objective:**

This study aimed to explore the properties of passively recorded environmental audio from a subject’s smartphone to find potential correlates of symptom severity of social anxiety disorder, generalized anxiety disorder, depression, and general impairment.

**Methods:**

An Android app was designed, together with a centralized server system, to collect periodic measurements of the volume of sounds in the environment and to detect the presence or absence of English-speaking voices. Subjects were recruited into a 2-week observational study during which the app was run on their personal smartphone to collect audio data. Subjects also completed self-report severity measures of social anxiety disorder, generalized anxiety disorder, depression, and functional impairment. Participants were 112 Canadian adults from a nonclinical population. High-level features were extracted from the environmental audio of 84 participants with sufficient data, and correlations were measured between the 4 audio features and the 4 self-report measures.

**Results:**

The regularity in daily patterns of activity and inactivity inferred from the environmental audio volume was correlated with the severity of depression (*r*=−0.37; *P*<.001). A measure of sleep disturbance inferred from the environmental audio volume was also correlated with the severity of depression (*r*=0.23; *P*=.03). A proxy measure of social interaction based on the detection of speaking voices in the environmental audio was correlated with depression (*r*=−0.37; *P*<.001) and functional impairment (*r*=−0.29; *P*=.01). None of the 4 environmental audio-based features tested showed significant correlations with the measures of generalized anxiety or social anxiety.

**Conclusions:**

In this study group, the environmental audio was shown to contain signals that were associated with the severity of depression and functional impairment. Associations with the severity of social anxiety disorder and generalized anxiety disorder were much weaker in comparison and not statistically significant at the 5% significance level. This work also confirmed previous work showing that the presence of voices is associated with depression. Furthermore, this study suggests that sparsely sampled audio volume could provide potentially relevant insight into subjects’ mental health.

## Introduction

### Background

Depression and anxiety disorders are some of the most prevalent mental health disorders [1], yet access to services and treatment for these disorders is lacking. It is common for many Ontario residents with mental health problems to wait for 6 months to 1 year for treatment [2]. Automation of a part of the mental health care process may help address this service gap in our health care system, as automated assessment could alleviate some of the workload that is currently being carried by health care workers.

The health care process can be modeled as beginning with assessment and measurement, followed by diagnosis, and finally treatment. Subsequent rounds of measurement or assessment occur with the final goal of achieving remission. This work focuses on the measurement and diagnosis components by working toward building an automated and objective severity measurement of anxiety, depression, and functional impairment associated with poor mental health.

Research in both psychiatry and clinical psychology traditionally involves assessments of subjects’ state (eg, mood and behavior) in clinical or research settings where they are removed from their natural home and living environment. Often, these assessments were performed retrospectively, where the subjects were asked to recollect behaviors and feelings over several weeks in the past. Ecological momentary assessment (EMA) [3] is an alternative approach that endeavors to assess subjects’ mood and behaviors in a naturalistic setting. It occurs in real time and has a higher frequency of measurement, possibly multiple times a day. These assessments are frequent and occur in subjects’ natural setting(s), removing clinicians (and the potential for bias), from the measurement process. Furthermore, EMA is not limited to self-report data but can also use data collected from sensors (physiological sensors and smartphone sensors). Sensor-based data are especially interesting in an EMA context because they can be collected *passively* without any interaction from the study subject, essentially addressing concerns of self-report biases surrounding self-reported data [4,5]. Researchers now use the term *passive EMA* [6] to refer to EMA systems in which sensor-based data are collected without any interaction from the user. It is also sometimes referred to as *unobtrusive* EMA or *passive sensing*.

The feasibility of building passive EMA systems has been greatly improved by the smartphone revolution. Smartphones are ubiquitous and affordable consumer electronics, which are equipped with a wide range of sensors that can enable the type of sensing or monitoring necessary to perform passive EMA [7]. A brief survey of some of the existing work using passive EMA in the mental health space follows, including a more detailed review of studies that have used ecological audio data to predict mental health state.

### Previous Work

A general methodology in these passive EMA or mobile sensing studies, and one that is used in this work, is to compute metrics, or *features,* from objective data sources that are designed to capture behaviors or traits of subjects that are known or suspected to be predictive of mental state. These features condense a large number of data points from a data source (eg, thousands of GPS coordinates measured over weeks of a study) into a single metric of behavior. This metric of behavior can then be tested for correlation with clinical measures of subjects’ mental state. One such example of a GPS location–derived feature is the proportion of time a subject spends outside home. A low proportion might be indicative of avoidance behavior or low energy and, therefore, relevant to depression, for example.

A systematic review by Rohani et al [8] examined correlations between passively sensed smartphone data and symptoms of depression. *Homestay*, the proportion of time spent by the subject at home (computed from GPS data), and *screen active duration*, the proportion of time spent using the phone were 2 of the most strongly correlated features with depression. These features were reported as significant by numerous studies included in the review [8].

This general methodology of sampling objective data from subjects’ smartphones (or other digital sensors) to infer health characteristics has been used in numerous studies, across many conditions. Although we will provide a focused review of relevant works that have used audio data to predict or measure mood and anxiety disorders, there is a wealth of research that has looked at using many different data sources to investigate, predict, or measure the severity of many characteristics of health and mental health disorders. Interested readers are directed to work that has investigated subjects’ general mood and mental health [9-15], substance abuse [16,17], depression [18-24], bipolar disorder [25-29], anxiety disorders [30-32], and schizophrenia [33,34]. The most commonly used sources of passively collected smartphone data in these works include subjects’ geolocation (ie, GPS data), screen activity and phone usage time, SMS and phone metadata, and physical activity and motion sensor data.

As all smartphones are equipped with microphones, they can be used to detect audio-based features of a subject’s environment. Several works have investigated the recording and analysis of speech audio from subjects’ smartphones. There are different strategies to record audio, ranging from (1) actively prompting users to speak into a microphone, (2) passively recording subjects’ phone calls, and (3) to passively recording environmental audio with no interaction from the user.

Using the active prompt-style methodology, Dickerson et al [35] conducted a study of depression in which subjects were asked twice daily to respond verbally to a prompt in free-form speech recorded by a microphone, yielding responses that were, on average, 1 to 2 min long. These audio recordings of prompted speech were then analyzed to produce 2 features: the fundamental frequency of subjects’ speaking voices (F_0_) and subjects’ speech pause time. These 2 features were used to build a linear model for predicting the mood of the subjects. Mood was measured on a 1 to 10 scale (on the continuum of extremely depressed to extremely elevated mood), and the linear model was able to predict mood scores with a residual error of 0.092 (12 degrees of freedom, *P*=.011) [35]. Similarly, Guidi et al [36] investigated the fundamental frequency of speech (F_0_) actively recorded from subjects via prompts in a study of bipolar disorder. This study was able to distinguish between individual bipolar subject’s mood states. A total of 7 features extracted from speech audio were subjected to Kruskal-Wallis tests, and all features showed significant differences (at a 5% significance level) across angry, neutral, bored, and happy emotional states.

Using the passive phone call recording–style methodology, Faurholt-Jepsen et al [29] conducted a study of 28 outpatient subjects with bipolar disorder. Voice features produced from patients’ phone calls were used to build 2 classification models that classified patients’ states as manic or mixed versus euthymic (area under the curve=0.89) and depressive versus euthymic (area under the curve=0.78). Another study of bipolar subjects conducted by Grünerbl et al [25] used features of subjects’ voices produced from the recordings of phone calls to predict mood state with 70% accuracy.

Finally, audio can be sampled in a much more passive and pervasive manner by using a smartphone’s microphone to record environmental (ambient) audio. The StudentLife study by Wang et al [37] sampled ambient audio and used audio analysis techniques to detect the presence of human voices in the environment as a proxy measure of conversation frequency. They found that conversation frequency has a significant negative correlation with self-reported measures of depression severity [37]. The work by Abdullah et al [26] used the same approach in a study of 7 subjects with bipolar disorder. The conversation frequency feature was found to be weakly correlated with mood patterns (*r*=0.16; *P*=.06) as measured by the social rhythm metric. Ben-Zeev et al [10] used the amount of time proximal to human speech as a predictive feature in a study of general mental health. In a functional regression analysis, the amount of time spent proximal to human voices was found to be significantly associated with changes in a self-reported measure of depressive symptoms over the course of the study (*P*=.048).

### Goal of This Study

This exploratory study seeks to discover potential correlates of anxiety and depression symptomatology from environmental audio acquired using passive smartphone sensing. Although previous research has studied how some features of the audio environment relate to depression and bipolar mood disorders, we will extend this to include anxiety disorders. In addition, one aspect of our study is the exploration of the sampled *average volume of the environment* over time, which has privacy-preserving attributes. We are not aware of any other study of mental health that makes use of this measurement. We hypothesize that the time series of the volume of subjects’ environments reveals some qualities and characteristics of their daily activities, which are associated with symptoms of depression and anxiety, a hypothesis that we believe has not been investigated in the literature. In addition, we explore the effect of the presence of voices in the sampled audio.

## Methods

### Overview

Subjects from a nonclinical population were recruited for a 2-week observational study in which a custom app was installed on their personal Android phone. Self-report measures of anxiety, depression, and general quality of life and impairment were collected at the beginning and end of the study. Throughout the duration of the study, the smartphone app passively collected the average volume of environmental audio and the presence of voice activity (whether or not speech was detected in the environment at the time of recording). A set of features was designed and used to extract higher-level information from this set of data, and a statistical analysis was performed to determine if a significant relationship existed between subjects’ self-reported anxiety, depression, and general impairment and these features. The study was approved by the University of Toronto Health Sciences Research Ethics Board (Protocol #36687).

### Recruitment

Subjects were recruited from Prolific [38], a web-based platform for recruiting study participants. Prolific maintains an active pool of subjects who wish to engage in research activities and enables researchers to deploy web-based tasks to subjects with specified demographics. It is similar to other services, such as Amazon’s Mechanical Turk [39], but has some properties that make it more attractive to academic researchers. These include ethical payment requirements and a comprehensive database of subjects’ demographic data to enable prescreening.

The study inclusion criteria were as follows: subjects should (1) reside in Canada, (2) be fluent in English, (3) own an Android phone, (4) have completed at least 95% of their previous Prolific studies successfully, and (5) have previously participated in at least 20 Prolific studies. The final criterion was used to ensure that subjects were proficient at using the Prolific system and were generally technology literate. There were no exclusion criteria for the study. Subjects were paid Can $18.50 (US $14) for participating in the study.

### Study Procedure

Members of the Prolific community who met the inclusion criteria could read a description of the study, which included an informed consent guide. Those who consented to the study were then directed to a webpage that acted as the study entry point. This website directed subjects to install the app from the Google Play app store and provided them with log-in credentials for using the study app. Once installed, the study app guided subjects through a short setup, where they were asked to provide the app with the necessary permissions to access their data, followed by a log-in. Immediately following setup and log-in, subjects were asked to complete a set of 4 self-report measures in digital form within the study app. At this point, following the completion of the self-report measures, the app began to periodically collect data in the background. No further actions or interactions with the study app were performed until the end of the study, exactly 14 days later, at the same time of day as the app installation/self-report work. At this time, subjects received a notification on their phone, informing them that the study had ended and requesting that they complete the same set of 4 self-report measures done at the beginning, again in the smartphone app. Following completion of this task, subjects were directed to uninstall the app from their phone and mark their study tasks as complete on the Prolific website. Subjects were then paid through Prolific’s payment system.

### Self-Report Measures

Subjects completed 4 self-report measures in digital form within the study app at the beginning and end of the 14-day study. A review by Belisario et al [40] found that self-administered survey scores do not differ when deployed by app versus other delivery modes. The 4 measures were the Liebowitz Social Anxiety Scale (LSAS) [41], the 7-item Generalized Anxiety Disorder Scale (GAD-7) [42], the 8-item Patient Health Questionnaire Scale (PHQ-8) [43], and the Sheehan Disability Scale (SDS) [44]. The LSAS is a 24-item scale used to assess the symptoms of social anxiety disorder by measuring respondents’ fear and avoidance of various social situations [41]. The LSAS was originally developed as a clinician-administered instrument, yet the self-report version has been shown to have good psychometric properties [45]. The GAD-7 is a 7-item self-report scale used as a screener and severity measure of generalized anxiety disorder [42]. The PHQ-8 is an abbreviated form of the Patient Health Questionnaire 9-item depression scale [46], which omits the final item of the PHQ-9, a question that assesses suicidal ideation. The PHQ-8, similar to the PHQ-9, has been shown to be a valid diagnostic and severity measure for depressive disorders [43]. The PHQ-8 was chosen instead of the PHQ-9 because, owing to the anonymous and remote nature of the data collection in this study, the study investigators would be unable to properly intervene in the case of any evidence of risk for self-harm. Finally, the SDS is a 3-item self-report measure of general impairment, which has been shown to be a sensitive tool for measuring mental health–related functional impairment [44].

Both the GAD-7 and PHQ-8 instruments ask subjects to evaluate their symptoms over the past 2 weeks, whereas the LSAS and SDS ask subjects to evaluate their symptoms over the past week. Therefore, 2 weeks was the shortest duration possible to encompass the largest time window of assessment of the self-report measures, which is the rationale behind a 2-week study duration.

### Smartphone Data Collection

An Android app was designed and created to collect all study data. This includes both the self-reported measures, described earlier, and the passively collected audio data—the volume of environmental audio and the presence or absence of speaking voices in the environment.

The study app records audio every 5 min, for a duration of 15 seconds, by turning on the microphone and recording the environment. This recording process occurs without any interaction from the user and with no notification to the user. Audio recordings are then securely transmitted from subjects’ smartphones over the internet to a computer server where 2 further processing steps are performed. First, the average volume of each 15-second audio recording was calculated using the FFmpeg audio processing software framework [47]. Second, the presence of voices in the audio recording was detected using the Google Cloud Speech-to-Text software product [48]. This software generates text transcripts from audio recordings of speech; it was used to detect the presence of speech in audio recordings by simply noting whether each audio recording generated a transcript. Audio files containing silence, noise, or unintelligible speech do not successfully generate a text transcript, whereas recordings that contain intelligible speech produce a transcript.

The audio sampling period was chosen to be 5 min as a good trade-off between large amounts of data (with a shorter period) and the preservation of battery life of subjects’ smartphones (with a longer period). Internal testing before the study showed a 5-min sampling period to be satisfactory for preserving battery life. Although a shorter period could yield more data, versions of the Android operating system since version 6 prevent this. Specifically, devices are prevented from performing background processing (such as this type of microphone sampling) while the device is sleeping more than once in a 9-min period [49]. Thus, for many devices, we are already at the limit of how frequently we can sample data in the background.

### Data Preprocessing

Preprocessing of the volume time series was performed before feature extraction to account for missing data and to perform normalization. Periodic audio recordings were not reliably produced at a precise period of 5 min by the study app, so the volume time series were resampled to a period of 5 min, and missing samples were imputed using linear interpolation. After resampling and interpolation, volume samples were clipped at the ceiling and floor of 3 SDs from the mean of the volume time series to remove outliers (using subject mean and subject SD, not group). Finally, the volume time series were scaled linearly to ensure that all volume measurements were within the range of 0 to 1. No preprocessing of the voice presence time series was performed.

### Feature Extraction

This subsection describes the methods used to compute the 4 correlates of anxiety and depression symptomatology derived from subjects’ environmental audio recordings. These correlates, or *features,* were extracted from the volume and speech presence time series data to test for the association with symptoms of anxiety and depression as measured by the LSAS, GAD-7, PHQ-8, and SDS. In the sections below, we describe 3 features from the volume time series, called *daily similarity*, *sleep disturbance-all nights*, and *sleep disturbance-weeknights*. A fourth feature was extracted from the speech presence time series called the *speech presence ratio*.

#### Daily Similarity

**Figure 1:**
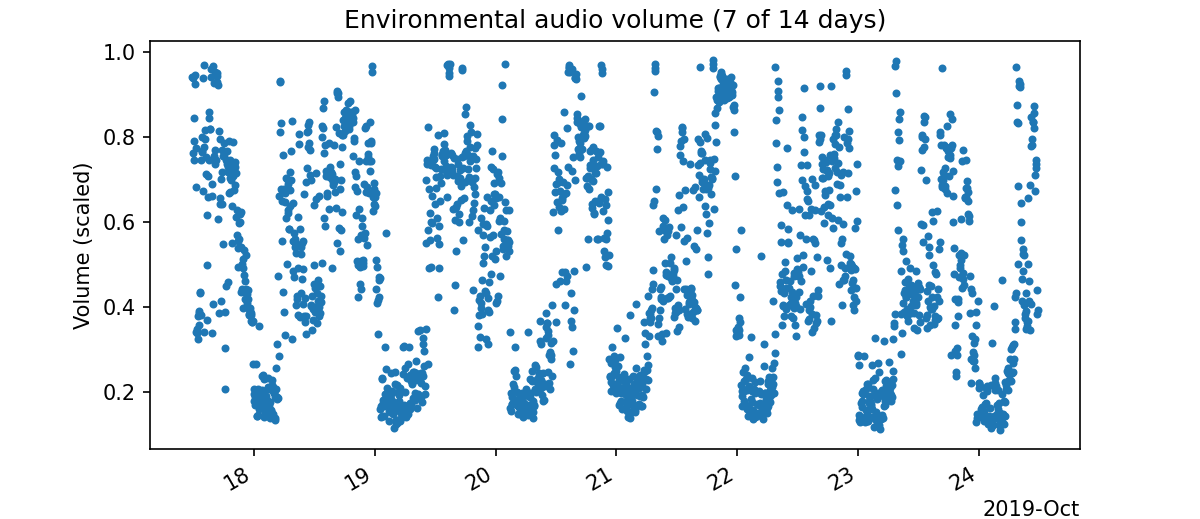
Visualization of a subject's environmental audio volume data (7 of 14 days).

The daily similarity feature was designed to infer the consistency of the subjects’ sequence of daily activities. Visualizations of the volume time series clearly show distinct periods of activity (characterized by large spikes in volume) and inactivity (characterized by quieter volume with less variance). These periods coincide roughly with daytime and nighttime, respectively. Furthermore, these patterns are periodic and repeat daily. Figure 1 shows a visualization of 7 days of a subject’s environmental audio volume data.

A link between regularity in daily activities, including sleep, and anxiety and depression is commonly described in the literature [50,51], and therefore, it follows that this feature may be associated with symptoms of anxiety and depression. To quantify the regularity of this pattern of daily activity, the *autocorrelation* function was computed for each subject’s volume time series. This is a signal processing technique that computes the correlation between a signal and a time-delayed copy of itself [52]. The autocorrelation function of a signal is a time-dependent Pearson correlation coefficient of the signal and its copy for varying degrees of time lag between the two. As we are interested in quantifying the similarity between a subject’s days, the value of the autocorrelation function at a time lag of 24 hours is most relevant. The *daily similarity* feature is, therefore, defined as the value of the autocorrelation function of the volume time series evaluated at a lag time of 24 hours.

#### Sleep Disturbance

Majority of the periods of apparent inactivity that are visible in the volume time series appear to coincide with sleep. It was hypothesized that a proxy measure of sleep quality can be inferred by measuring how chaotic the volume of subjects’ environments are during sleep times, replicating the link between sleep and mental health reported in the literature. For example, the prevalence of mood disorders has been shown to be much higher in populations with chronic sleep problems [53], and insomnia may be a state marker of anxiety disorders [54]. It follows, therefore, that a feature that infers the quality of subjects’ sleep may be associated with symptoms of anxiety and depression.

To quantify sleep quality, the volume time series was examined with periods of quiet noted to be characterized by low variance in volume. Although the absolute value of the volume is also low at quiet times, the threshold for what can be considered quiet is greatly dependent on the specific microphone and phone placement; therefore, variance was considered a more appropriate measure of the noisiness of the environment. The *sleep disturbance* feature is defined as the SD of the volume time series between the hours of 12:00 AM and 06:00 AM, local time. This is a proxy measure of sleep quality. More specifically, it is a measure of the noisiness of a subject’s environment during common hours of sleep. In addition, as people’s patterns of sleep can vary between weeknights and weekends, we compute 2 versions of this feature: (1) using volume data between the hours of 12:00 AM and 06:00 AM on all nights of the week, and (2) using only volume data from weeknights (Monday through Friday). These choices are made without specific knowledge of the subjects’ workday or workweek schedule.

#### Speech Presence Ratio

Previous studies of mental health using mobile sensing have computed proxy measures of social interaction as a feature predictive of depression severity [10,37]. To replicate these results and to ascertain if this also holds for measures of social anxiety, generalized anxiety, and general impairment, a proxy measure of social interaction was computed. The *speech presence ratio* feature is computed from the speech presence time series data and is defined as the proportion of audio recordings containing the presence of speech to the total number of audio recordings.

### Privacy

A number of privacy considerations drove the design of the study procedure, app, and data collection. Prolific, the platform from which subjects were recruited, anonymizes subjects. Subjects were provided with app log-in credentials, which were provided on demand to each subject as they enrolled in the study to avoid subjects using their name, email address, or some other potentially identifying information as their log-in name. Audio recordings were encrypted both at rest (on subjects’ phones) and in transit to the server. Once processed on the server side, audio files were deleted. The speech transcripts generated to detect the presence of speech were processed in the following way: each transcript was broken into pairs of words (ie, bigrams) and then stored in random order for use in later studies. The stripping of ordering and time information from bigrams was done to prevent later re-creation of transcripts.

## Results

### Overview and Data Inclusion

**Figure 2:**
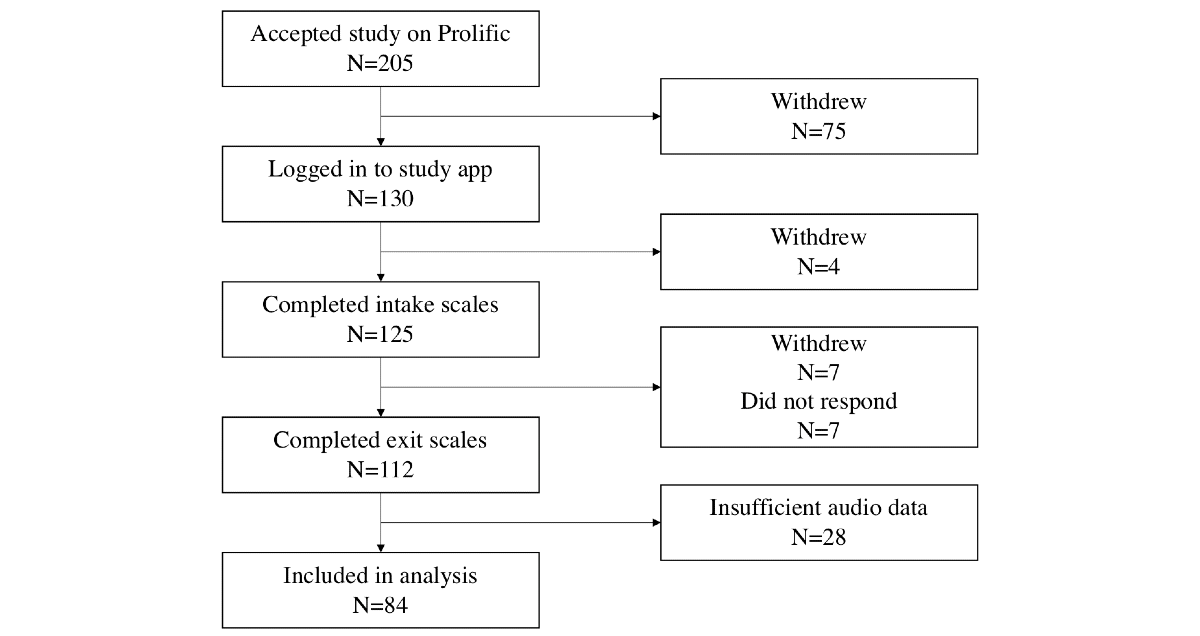
Flow chart of study recruitment.

From July 2019 to December 2019, 205 eligible Prolific members entered the study. Withdrawals were common, with 86 subjects choosing to withdraw at some point in the study (commentary on the high withdrawal rate is provided in the Limitations subsection of the Discussion section). Of the 119 subjects who did not withdraw, 112 completed both sets of self-report questionnaires. Finally, 84 of the 112 completed subjects yielded sufficient audio data for analysis based on the criterion that at least 50% of the ex*p*ected number of audio recordings were made by the study app. Figure 2 provides an illustration of the study recruitment results. Although self-report measures were collected at both the beginning and end of the 2-week study, all results that followed used the values collected at the end of the study. The postobservation values of the self-report measures were used in the analysis because if such a smartphone-based assessment system were to exist, it would be more useful to predict trajectories or upcoming symptom severity, not severity 2 weeks before the start of data recording (as would be the case if correlations/associations with the prestudy scales were measured).

### Study Group Characteristics

The study sample had an average age of 30 years (SD 8.6) and 42% (35/84) of subjects were female. The mean and SD of the 4 self-reported measures are presented in Table 1. To understand whether the self-reported measures may be related to the age and sex of subjects, 2 statistics were computed. The Pearson correlation coefficient was computed to measure the correlation between self-reported measures and subject age, and an independent samples *t* test was performed to test whether the mean scale score differed between the male and female sexes. The LSAS, GAD-7, and SDS were negatively correlated with subject age, but there was no significant difference in the mean scale scores of the 2 sexes (at a 5% significance level). Table 1 also lists the results of these tests.

To further characterize the mental health of our study subjects, self-report measures were used to screen for social anxiety disorder, generalized anxiety disorder, and major depressive disorder. A cutoff of 60 was used with the LSAS scores to screen for social anxiety disorder (generalized subtype), as recommended by Mennin et al [55]. A cutoff of 10 was used with the GAD-7 scores to screen for generalized anxiety disorder. This cutoff was shown to optimize sensitivity (89%) and specificity (82%) [42]. A cutoff of 10 was used with the PHQ-8 scores to screen for depression, as recommended by Kroenke et al [43]. Table 2 summarizes the results of these screenings on our study sample. The rates of social anxiety (32%), generalized anxiety (26%), and depression (37%) in our study sample were all compared with the rates in the general Canadian population. This will be elaborated in the Discussion section.

**Table 1:** Descriptive statistics for self-report measures of anxiety and depression (n=84).

Measures	Score, mean (SD)	Correlation with age		Difference between mean scores of the sexes	
		*r*	*P* value	*t* test (*df*)	*P* value
Liebowitz Social Anxiety Scale	53.7 (25.8)	−0.27	.01	−1.68 (82)	.10
Generalized Anxiety Disorder seven-item scale	6.6 (4.6)	−0.29	.01	−1.37 (82)	.17
Patient Health Questionnaire eight-item scale	8.5 (5.6)	−0.19	.09	−1.18 (82)	.24
Sheehan Disability Scale	10.8 (7.7)	−0.26	.02	−1.12 (82)	.27

**Table 2:** Results of screening the study sample for depression and anxiety disorders (n=84).

Disorders	Screening criteria	Positive screenings, n (%)
Social anxiety disorder	Liebowitz Social Anxiety Scale score ≥60	32 (38)
Generalized anxiety disorder	Generalized Anxiety Disorder seven-item scale score ≥10	22 (26)
Major depressive disorder	Patient Health Questionnaire eight-item scale score ≥10	31 (37)

### Objective Audio Features

The objective audio features described in subsection *Feature Extraction* of the Methods section were computed using the environmental audio time series data of the 84 study subjects. Descriptive statistics for the 4 features are presented in Table 3. These descriptive statistics include the mean and SD of the features observed in the study sample, and additionally the minimum value observed, the maximum value observed, and the values of the first three quartiles. The values of the speech presence ratio appear to align reasonably with intuition, with subjects spending anywhere from 1% to 30% of their time in the presence of speech.

**Table 3:** Descriptive statistics for objective audio features (n=84).

Features	Mean (SD)	Minimum	Q1	Q2	Q3	Maximum
Daily similarity	0.80 (0.07)	0.45	0.77	0.83	0.85	0.90
Sleep disturbance—all nights	0.14 (0.06)	0.03	0.10	0.13	0.17	0.32
Sleep disturbance—weeknights	0.14 (0.06)	0.03	0.09	0.12	0.18	0.34
Speech presence ratio	0.15 (0.06)	0.01	0.11	0.16	0.20	0.30

### Correlations Between Audio Features and Self-Report Measures

To test the association between the audio features and the self-reported measures of anxiety, depression, and functional impairment, the Pearson correlation coefficient was computed between each feature and scale. The daily similarity feature is negatively correlated with all 4 self-report measures, which supports the hypothesis that regularity in daily activity and circadian rhythm is associated with more positive mental health (ie, lower scale scores). This feature was most strongly correlated with depressive symptoms. The sleep disturbance feature, whether computed using all nighttime audio or only weeknight audio, was positively correlated with all 4 self-report measures, which is in line with the hypothesis that better sleep quality (ie, less sleep disturbance) is associated with positive mental health. The strength of the correlation is improved when only the weeknight audio is considered. Finally, the speech presence ratio feature was negatively correlated with all 4 self-report measures, where the correlation with depressive symptoms was the strongest for all observed correlations (*r*=−0.37; *P*<.001). Table 4 summarizes the results of the correlation analysis. No correction for multiple tests was performed, as we view this work as exploratory.

**Table 4:** Pearson correlation between objective audio features and self-reported measures of anxiety and depression (n=84).

Features	Liebowitz Social Anxiety Scale		Generalized Anxiety Disorder seven-item scale		Patient Health Questionnaire eight-item scale		Sheehan Disability Scale		
	*r*	*P* value	*r*	*P* value	*r*	*P* value		*r*	*P* value
Daily similarity	−0.20	.07	−0.19	.09	−0.37	<.001		−0.18	.10
Sleep disturbance—all nights	0.00	.99	0.07	.52	0.17	.13		0.15	.17
Sleep disturbance—weeknights	0.05	.65	0.12	.26	0.23	.03		0.18	.11
Speech presence ratio	−0.19	.08	−0.16	.14	−0.37	<.001		−0.29	.01

## Discussion

### Population

The self-report measures completed by the subjects revealed that this study’s sample, despite being recruited from a healthy population, had a high prevalence of depression and anxiety. Data reported by the Government of Canada in 2006 estimate a 12-month prevalence of major depressive disorder at 4.8% [1], compared with a positive screening rate of 37% in our sample. The same 2006 report lists a 12-year prevalence of the combined class of anxiety disorders at 4.8%, a figure that is significantly lower than the positive screening rates for generalized anxiety disorder (26%) and social anxiety disorder (38%) observed in our sample. Given that the cutpoints used for screening were all shown to have high specificity, it seems unlikely that these high rates are solely a result of false-positive screenings. Instead, 2 possible explanations are proposed, which may be jointly responsible. First, the Canadian population of subjects on the Prolific recruitment platform may have elevated rates of mood and anxiety disorders with respect to the general Canadian population. It may be that people who choose to find work on the platform go there because of these conditions, which might prevent them from doing outside-the-home work. Second, that subject sampling was impacted by self-selection bias. In other words, Prolific participants who struggle with mental health to some degree may be more likely to have chosen to enroll and remain in a study that focuses on mental health.

Negative correlations were measured between age and self-reported measures of social anxiety (*r*=−0.27; *P*=.01), generalized anxiety (*r*=−0.29; *P*=.01), depression (*r*=−0.19; *P*=.09), and general impairment owing to poor mental health (*r*=−0.26; *P*=.02) in our sample of subjects. This indicates that younger subjects generally displayed worse mental health than older subjects. It is not clear if this observation is part of a more general trend in the greater population or if it is specific to the Prolific population. A review of studies examining the occurrence of anxiety, depression, and distress found some evidence that aging is associated with less susceptibility to anxiety and depression, but it is unclear if that was because of aging or cohort effects [56]. A study of mood disorders and suicide using data on American adolescents and adults from 2005 to 2017 observed an increase in mood disorders and suicidal ideation since the mid-2000s, and it is suggested that cultural trends in the use of digital media among younger individuals may be responsible for creating a cohort effect [57].

### Correlations

The key finding of this work is the development and evaluation of a set of features, computed from subjects’ environmental audio, as potential correlates of symptoms of anxiety, depression, and functional impairment. Correlation analysis of these features and self-reported measures, summarized in Table 4, shows that all 4 features are associated more strongly with symptoms of depression (measured by the PHQ-8) than with any other symptoms. The daily similarity and speech presence ratio were both significantly correlated with PHQ-8 scores at a 5% significance level. Speech presence ratio, but not daily similarity, was likewise significantly correlated with SDS scores at a 5% significance level. None of the 4 features were found to be significantly correlated with either LSAS or GAD-7 scores at a 5% significance level.

We note that while associations with the LSAS and GAD-7 are weak or nonexistent, the associations with the SDS are nearly as strong as those with the PHQ-8. This may suggest that the impairment that is being measured by the SDS may, in large part, be due to symptoms of depression. Indeed, we measured a stronger correlation between the SDS and the PHQ-8 scores of subjects (*r*=0.76; *P*<.001) than between the SDS and the LSAS (*r*=0.48; *P*<.001) or the GAD-7 (*r*=0.62; *P*<.001).

The fact that some features are associated with depressive symptomatology but none are associated with the symptomatology of generalized anxiety or social anxiety disorder is interesting, and we offer some speculation as to why this may be the case. First, we must observe that our features are very coarse—they measure sleep, activity, and speech, but with no specific context (they are measured on a gross scale). Depression is broadly debilitating on energy and activity, and if this impact is independent of a specific context, our features are appropriately designed to detect this impact. This is in contrast to anxiety disorders, which often have specific triggers that are context dependent. Individuals with anxiety can avoid contexts that trigger their anxiety and, therefore, present as if they do not suffer from the effects of anxiety as long as they continue to exhibit avoidance behaviors. Although it is unlikely that anxious individuals are able to avoid all triggers, especially those with generalized subtypes, avoidance behavior may be partially responsible for weakening the associations between inferred behavior and mental state. To passively measure the severity of anxiety disorders, it seems that any feature must capture avoidance behavior as a proxy for anxiety itself and also have some measure of state anxiety to detect when a subject is in a specific context that acts as an anxiety trigger.

### Comparison With Other Studies

To our knowledge, no other studies have inferred daily patterns of activity and inactivity solely from volume samples of ambient audio, which is captured by the daily similarity feature, so direct comparisons of the daily similarity feature with other known works are not possible. However, a feature called circadian movement, first proposed by Saeb et al [20], captures the degree to which the pattern of a subject’s visits to different geolocations follows a 24-hour or circadian rhythm. The study by Saeb et al [20] measured a significant negative correlation between circadian movement and PHQ-9 scores (*r*=−0.63; *P*=.01; n=18). Our ambient volume-based measure of daily regularity in activity, called daily similarity, was also negatively correlated with PHQ-8 scores (*r*=−0.37; *P*<.001; n=84). This further supports the hypothesis that regularity in daily activities is associated with lower severity of depressive symptoms. It is interesting to consider that the same underlying signal may be present in both GPS data and ambient audio data. A study by Ware et al [58] also used the circadian movement feature in the prediction of depression. Although this feature was one of many used in a classifier that achieved an F1 score as high as 0.86, it is unclear to what degree this feature alone was associated with the severity of depressive symptoms.

The association between sleep and mental health has been investigated in a number of studies. Self-reported measures of sleep quality have been shown to be associated with state anxiety [59], anxiety disorders [60], and depression [61,62]. Sleep duration has been measured objectively in mobile sensing studies and shown to have a significant association with the severity of depressive symptoms [10,23,37]. although sleep duration and sleep disturbance features are different measures and as such cannot be directly compared, these studies support our results in demonstrating a general association between sleep and depression.

Finally, although the speech presence ratio feature does not appear in identical form in the literature, there are other studies that have used similar proxy measures of social interaction as correlates of depression severity. The pioneering study by Wang et al [37] inferred the number of conversations that subjects encountered throughout their days by performing an analysis of ambient audio and also found a significant negative correlation with PHQ-9 scores (*r*=−0.39; *P*=.02; n=48). This specific feature by Wang et al is more contextually aware than our speech presence ratio because not all audio that they found to contain speech was considered conversational in their system. Specifically, they ignored speech if it was detected during lecture or group meeting hours of their student subject population. Despite the fact that our proxy measure of social interaction is context unaware, we also show a negative correlation between the speech presence ratio and PHQ-8 scores (*r*=−0.37; *P*<.001; n=84). Time spent proximal to human speech was also found to be significantly associated with changes in PHQ-9 scores (*P*=.048) in a study by Ben-Zeev et al [10].

### Limitations

A fundamental limitation of the study design is that as a cross-sectional study, it is not possible to make any claim regarding causation between the observed features and severity of anxiety, depression, or functional impairment. It cannot be determined, for example, if avoiding social contact (as inferred by the speech presence ratio feature) causes increased depression severity or if an increase in depression severity owing to some other factor causes individuals to retreat socially and engage in less speaking.

A high proportion of subject withdrawals can also be considered as a limitation of this study (86/205 subjects, 42%, chose to withdraw). The majority of withdrawals, 87% (75/86), occurred before a successful log-in to the study app (see Figure 2 for an illustration of the withdrawals throughout the study timeline). Having observed how most withdrawals occurred early in the study, we provide 2 possible hypotheses for the high number of withdrawals. The first is the relative difficulty in setting up the study app on a personal smartphone, which is more complex than the typical task asked of subjects recruited on the Prolific platform. Our setup procedure included turning off the smartphone’s battery optimizations for the study app, which requires some facility with Android settings. The second is the possibility that subjects are unwilling to provide the app with the permissions necessary for data collection. The study app asks users to grant permissions before log-in, which might explain why 87% (75/86) of the withdrawals occurred without logging in. Our previous study explored clinical patients’ willingness to consent to the collection of different forms of data collection for mental health purposes [54]. The number of withdrawals is in line with the data from the previous study.

A further limitation surrounding subject withdrawals is the possibility of sample bias. The 86 individuals who withdrew from the study may differ from those who remained in the study. We are unable to test this because Prolific removes researchers’ access to the demographic data (age and sex) of participants who withdraw from studies. Nondemographic data (ie, digital data and self-report measures) may also differ between the groups, yet this is difficult to test because 92% (79/86) of withdrawals withdrew early enough in the study so as not to provide any digital data or self-report measures. Of the 112 individuals who remained in the study and completed all tasks, it is possible to test for differences between the group included in the analysis (ie, the group with sufficient audio data) and the group excluded from the analysis. These 2 groups did not differ significantly in age or on any of the 4 self-report measures, either at intake or exit, as tested by *t* tests at a 5% significance level (two-sided). These 2 groups also did not differ by sex (counts of men and women), as tested by a chi-square test (1 degree of freedom) for independence at a 5% significance level.

Some limitations also exist regarding the validity of the features. The speech presence ratio feature does not distinguish between recorded speech (eg, from a TV or radio) and human speech, it simply detects intelligible speech. This method does not distinguish between speakers, so in many cases, the subject themselves may not be the person speaking. The method also only detects English speech, so it will potentially miss speech if, for example, a subject does not speak English at home. Finally, our technique for detecting speech using automatic speech recognition is more biased toward false negatives than false positives. If speech is detected by the system, it is highly likely that the speech is present, yet it is much more likely to miss speech in noisy environments or environments with multiple speakers speaking concurrently.

The sleep disturbance feature is affected by the subjects’ specific mobile phone hardware, where different microphones with different automatic gain control functionality (which dynamically adjusts the volume and is not controllable by programmers) could produce different measurements in the same environment. To produce perfectly consistent features, one would be required to use a device such as a calibrated sound level meter, which measures volume as an absolute measure with no gain control.

Finally, it must be noted that the feasibility of completely passive mobile sensing with a high frequency of data sampling is becoming increasingly difficult on Android devices. Battery optimization features limit the rate at which apps can turn on in the background and sample their environment [63] because any app activity that *wakes up* a device not actively being used will drain device battery. Although Google does offer mechanisms for limiting battery optimizations to improve the frequency at which passive sensing can occur, many Android device vendors add third-party battery optimization software that can be difficult to disable systematically. This vendor-specific constraint on apps is a significant issue because of the fragmented nature of the Android ecosystem.

### Conclusions

This work contributes toward the development of automated and objective severity measurements of anxiety, depression, and functional impairment associated with poor mental health. Focusing solely on environmental audio, which was passively sensed from subjects’ smartphones, this work presents 2 new correlates of depressive symptoms and general impairment, which we refer to as the daily similarity and sleep disturbance features. Furthermore, this work supports previous findings by reproducing a measured association between time spent proximal to speech and severity of depression.

## Abbreviations

EMA: ecological momentary assessment
GAD-7: 7-item Generalized Anxiety Disorder Scale
LSAS: Liebowitz Social Anxiety Scale
PHQ-8: Patient Health Questionnaire eight-item scale
SDS: Sheehan Disability Scale
